# General Erethism Produced by Injury of the Membrana Tympani

**Published:** 1846-09

**Authors:** Joseph B. Cottman

**Affiliations:** Whitehaven, Md.


### MEDICAL EXAMINER

AND

### RECORD OF MEDICAL SCIENCE.

NEW SERIES.—No. XXI.—SEPTEMBER, 1846.

### ORIGINAL COMMUNICATIONS.

*General Erethism produced by injury of the Membrana Tympani.* By Joseph B. Cottman, M. D., of Whitehaven, Md. (in a letter to Prof. Dunglison.)

*March 28th,* 1846.—Mrs. J., on the night of the 28th, while picking her ear with a knitting needle, accidentally passed it in too far, so as to injure the membrana tympani; the effect of the injury was instantaneous; she seized hold of the nearest object to prevent her from falling from the chair, and called for assistance. With some difficulty she was carried to an adjoining room in a state of insensibility; being placed on a bed, she recovered her reason sufficiently in a little while to tell what had happened to her. *State at this time.*—Expression wild, pupils very much dilated, face flushed, the least motion of the head seemed to give the most excruciating pain; she would scream aloud; tetanic twitching of the muscles of the arm;—pulse strong, full and bounding; violent throbbing of the carotids. In the course of fifteen or twenty minutes, this state of things was succeeded by general syncope; her face would become blanched, her extremities cold, long and laboured respiration, with occasional sighing; this would continue for half an hour or more, before she could be aroused; when aroused, her conversation was incoherent, her face flushed, pupils preternaturally dilated, violent sick stomach, with occasional vomiting; rigors; extremities cold. This state of things continued alternately from 10 o'clock at night until 3

o'clock in the morning, when she fell asleep. Slept about three hours.

*March 29th,* 6 o'clock, A. M.—Still complained of pain in her head; the least motion aggravated it; said that her mind was very much confused, that she could not think; face flushed; pupils dilated; tetanic twitching of the muscles of the extremities; occasional flushes of heat and cold as she described it; pulse full, strong and corded; conversation at times incoherent, I tied up her arm, and took about a quart of blood with decided benefit; her pulse became natural; her mind clearer; talked more rationally; said that her head felt better, that she could hear a little in the injured ear. Up to this time she had not heard at all in that ear from the time of the accident. She felt so much better that she desired her female attendants to take her dress off; in attempting to do so she was placed in an upright position, this produced syncope which continued for nearly an hour; during this time her breathing was stertorous and labored; her extremities cold; occasional twitching of the muscles of the arm; pulse very slow and feeble; it was with the utmost difficulty that she could be aroused, and when aroused complained of being very chilly; violent sick stomach and a constant disposition to vomit. In the afternoon, two small blisters were applied behind the ears; these drew well, and produced a general amelioration of all her bad symptoms; she fell into a quiet sleep at night, and slept well until morning.

*March 30th,* 6 o'clock, A. M.—On awaking in the morning she had considerable fever; restless; thirst urgent; nausea with a disposition to vomit; about twelve o'clock the fever passed off, and she said she felt much better; could turn in bed without producing any unpleasant feeling about her head; mind clearer; talked more rational; expression better; thought she could hear better. In the afternoon she fell asleep, and slept till near night; at this time I left her; I saw her again about 9 o'clock, P. M.; at that time she was decidedly better than she had been; expression natural; talked rationally; says she is entirely free from pain.

*March 31st,* 6 o'clock, A. M.—Did not sleep well last night; return of fever, restlessness; thirst very urgent; craves ice; complains of a *roaring* in the injured ear like distant thunder; says that she sometimes loses her senses; cephalalgia very great, confined to the forehead; fever passed off about 10 o'clock, when she fell asleep; slept about an hour with decided benefit; says she always feels better after sleeping. In the afternoon I gave her eight grains of blue mass.

*April 1st,* 6 o'clock, A. M.—Slept well; fever very slight; a general improvement in her situation; slight *roaring* in the injured ear; blue mass has not operated; took half an ounce of calcined magnesia; this produced a gentle action on the bowels. From this time, she gradually convalesced without a return of any of her unpleasant symptoms, and is now perfectly restored.

JOSEPH B. COTTMAN, M. D.

*April 25th*, 1846.

